# Frequency and Associated Factors of Parental Refusal to Perform Lumbar Puncture in Children with Suspected Central Nervous System Infection: A Cross-sectional Study

**DOI:** 10.7759/cureus.5653

**Published:** 2019-09-14

**Authors:** Mushtaq Ahmed, Muzamil Ejaz, Ashraf Jahangeer, Sumaiya Khan, Syeda Shaheera Riaz Hashmi, Tabinda Jawaid, Saad Nasir

**Affiliations:** 1 Pediatrics, Civil Hospital Karachi, Dow University of Health Sciences, Karachi, PAK; 2 Epidemiology, Civil Hospital Karachi, Dow University of Health Sciences, Karachi, PAK; 3 Internal Medicine, Civil Hospital Karachi, Dow University of Health Sciences, Karachi, PAK; 4 Internal Medicine, Dr. Ruth Pfau Civil Hospital, Dow University of Health Sciences, Karachi, PAK; 5 Internal Medicine, Baqai Medical University, Karachi, PAK; 6 Internal Medicine, United Medical and Dental College/Creek General Hospital, Karachi, PAK

**Keywords:** lumbar puncture, pakistan, cns infections

## Abstract

Objective

Lumbar puncture (LP) is a useful procedure which is performed for both diagnosis and treatment of numerous conditions affecting children and adults. The purpose of this study was to determine the frequency and cause of increased parental refusal to perform LP in the pediatric population.

Method

A cross-sectional study was conducted from January 2018 to June 2019 at the Civil Hospital, Dow University of Health Sciences, pediatric department, Civil Hospital, Karachi. Over the 18-month time period, a total of 215 patients who had indications of LP were selected from the in-patient pediatrics department; the age range was between newborn to 12 years of age. The mode of research was a questionnaire and interview-based method that was conducted with guardians of minor patients to understand the extent of their knowledge and awareness about the LP procedure as well as its complication and the role of culture, education background, and financial status of the families which may lead to an increased likelihood of refusal.

Result

The frequency of LP refusal amongst the 215 families of the patients that were interviewed was found to be 32.6%. Mean age of the respondents was 30.98 years. The decision for LP was not significantly affected by the subjects’ gender (p=0.1), by the religious communities to which the families belonged (p=0.9), their ethnicities (0.52), or by the families’ financial status (p=0.4). It was observed that when indications for performing LP were appropriately explained, there was a significantly greater number of consents given as compared to when they were not made clear (p=0.009). Explaining the complications of the procedure did not considerably impact the decision for refusal of the procedure (p=0.1). The multi-variable logistic regression analysis model was applied to determine the likelihood of variables affecting refusal of LP and the logistic regression model was found to be statistically significant, χ2 (8) = 38.2 p < 0. 001.

Conclusion

Lack of knowledge about the LP procedure and fear of ramification plays a conspicuous role in the denial of LP procedure by the guardians of minor patients. A better, simpler approach using standardized consent forms by the doctors may lead to the removal of the information gaps and can provide a better understanding about the concerned risks, the primary indications, and the benefits of this procedure to the guardians.

## Introduction

Every society has its own set of religious and cultural beliefs and they play an important role in the refusal of various medical interventions such as lumbar puncture (LP). LP is frequently performed in the pediatric population but there is a high rate of refusal from parents for this procedure. A few studies have been conducted in this regard which showed refusal rate as high as 80% in Kuwait, 62% in Iran, 44% in the UAE, 24.7% in Malaysia, and 7% in Denmark narrowing down to 5% in the United States [1]. Several studies have been conducted to find the root cause of refusal to LP. According to a study performed in the UAE, fear of various complications was the reason behind the refusal of 43% of families where 11% of families thought that this procedure was unnecessary [2]. A similar study was conducted in Iran which showed that 67% of the families refused due to the perception of LP leading to fear of paralysis and backache (54.7%) [3].

Some studies showed an association of different ethnicities and level of education with higher refusal rates. A study in Iran revealed a major difference in refusal rates between Afghan and Iran ethnic group [4]. A study done in UAE showed that parents with higher knowledge of complications from bacterial meningitis gave consent more frequently than the ones who had lesser knowledge [2]. A similar study in Kuwait revealed that education and knowledge were the key factors that influenced parents’ decision regarding LP. The study found increased acceptance rates among parents with a higher level of education and higher knowledge scores [1]. It also revealed the importance of counseling, most parents (78%) changed their decision of refusal to LP after their doctor explained the procedure [1].

The refusal to LP usually results in empirical intravenous administration of antibiotics which increases the chances of antibiotic resistance and/or prolongs hospital stay and usually results in failure of administering prophylactic antibiotic therapy for contact bacterial meningitis [2]. To the best of the authors’ knowledge, statistical data on refusal to LP is still lacking adequately in Pakistan; therefore, in our study, we aimed to determine the frequency and risk factors for refusal to LP.

## Materials and methods

This was a cross-sectional study of children (newborn to 12 years of age) with signs and symptoms of infections of the central nervous system (CNS) whose parents were offered diagnostic LP and who were admitted in the pediatric department of Civil Hospital, Dow University of Health Sciences, Karachi from January 2018 to March 2019. Data was collected by means of a questionnaire survey for parents of children in whom LP was indicated and who gave informed consent for voluntary participation. In case of unavailability of parents during the interview, relatives were asked for consent and interviewed on the parents’ behalf. Children in the emergency department were not included in the study to minimize potential families’ response bias. Instead, the survey was conducted after admission in inpatient pediatric departments when the families’ anxiety had comparatively reduced and they were less likely to fear their physician’s partiality in management, should they refuse to participate.

The interview was conducted by trained personnel in the interviewee’s native language and, if required, translated by an interpreter. Due to cultural unacceptability, audio/video recordings were not brought into use. The interview was designed to assess knowledge, awareness, and perception of the community about diagnostic LP. Data was anonymized, comprising of unidentifiable elements of demographic and socioeconomic profiles including age, gender, ethnicity, religion, level of education and monthly income. Signs and symptoms that steered physicians towards offering LP as a diagnostic tool were recorded. The survey further detailed the interviewee’s perception and knowledge about complications that could occur from this procedure and thorough inquisitions were made to determine the reasons behind the family’s decision to permit or refuse LP.

Data was entered into and analyzed by means of Statistical Package for Social Science, version 22 (SPSS Inc., Chicago, IL) for frequency and percentage distribution. Chi-square test was employed to determine the association between the demographic variables and responses. P-value of less than 5% was considered as significant. The multi-variable logistic regression analysis model was used to determine the likelihood of variables affecting refusal to LP. Ethical approval to conduct the study was obtained from the Institutional Review Board of Civil Hospital, Dow University of Health Sciences.

## Results

**Figure 1:**
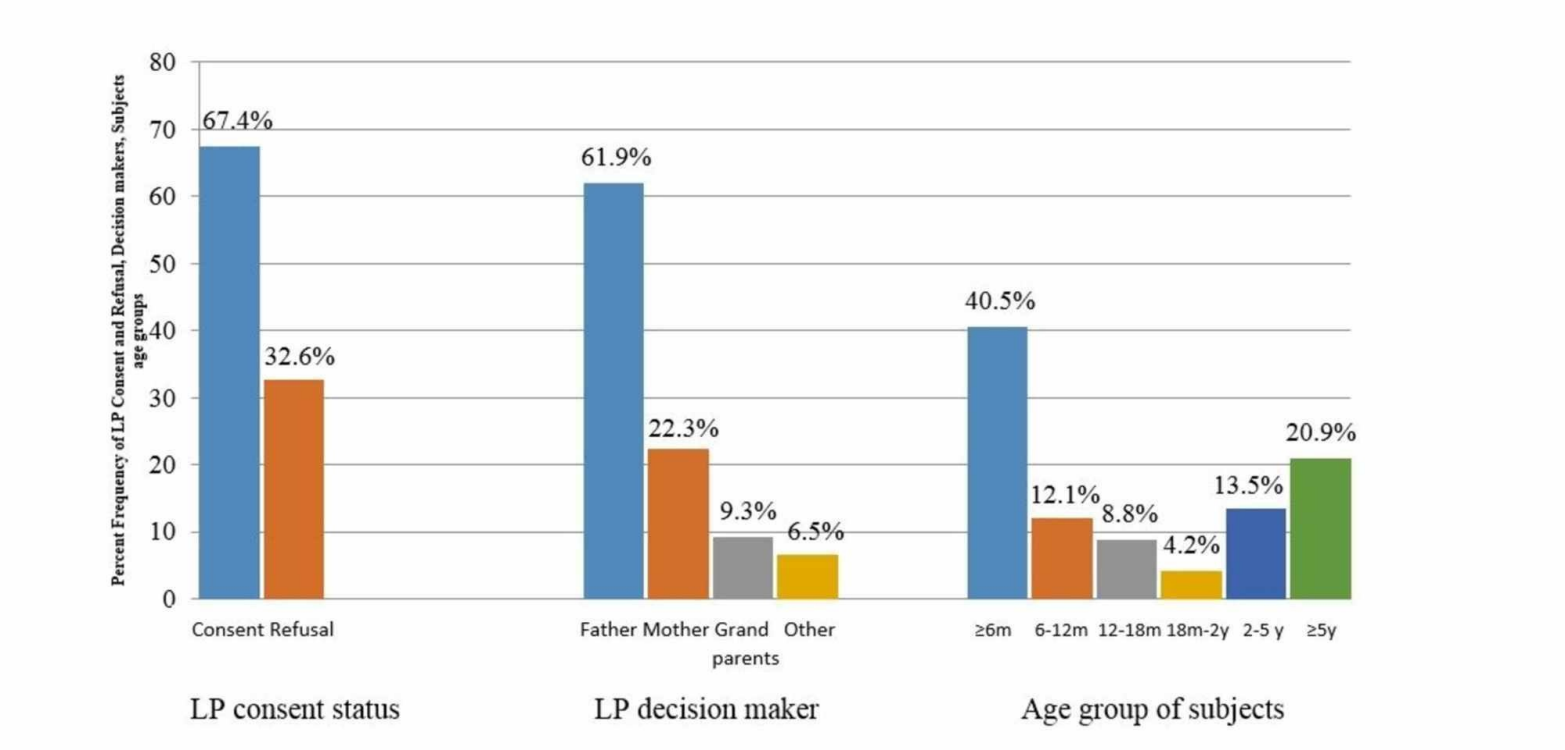
Lumbar puncture (LP) consent status, age group, and gender of subjects

Two hundred and fifteen patients between newborn to 12 years of age with LP indications were recruited over a period of 15 months. Amongst the 215 families interviewed, 145 (67.4%) of these gave consent for LP while 70 families (32.6%) refused the procedure. The subjects included 119 male patients (55.3%) and the mean age was 2.7 years, mostly falling under the age group of ≤ six months (40.5%) (Figure 1).

Although there was no significant discrimination based on gender regarding the decision to permit or refuse LP (p=0.16), it was interesting to note that the patients’ age group had a significant effect, with consent given for children younger than six months of age being higher as compared to older children (p=0.019). Parents and relatives who were interviewed consisted of mostly females, usually being the mother (58.6%). Mean age of the respondents was 30.98 years. However, the decision to permit or refuse LP was seen to be made majorly by fathers (61.9%) (as seen in Figure 1), regardless of the subjects’ ethnicities (p=0.2) and religious communities to which the families belonged (p=0.62). It was worthy of note that in cases where the children’s fathers made this decision, there was a significantly greater number of consents given for LP as compared to when the mothers or grandparents were the decision-makers (p=0.006).

The decision for LP was not significantly affected by the subjects’ gender (p=0.1), by the religious communities to which the families belonged (p=0.9), their ethnicities (0.52) or by the families’ financial status (p=0.4). The decision maker’s level of literacy, however, appeared to have a considerable effect (p=0.004). They were categorized into three groups: group 1 consisted of persons that had received no formal education, group 2 comprised of persons who had received either primary or secondary education, while group 3 contained persons with education beyond secondary school. Unexpectedly, group 1 and group 3 consented to LP more readily (76.2% and 70.2%, respectively) than group 2 (53.3%) (Table 1).

**Table 1:** Lumbar puncture status and literacy level

		Lumbar Puncture Status		Total
		Consent	Refusal	
Level of Education	None	93 (76.2%)	29 (23.8%)	122 (100%)
	Primary or below	40 (53.3%)	35 (46.7%)	75 (100%)
	Secondary or above	12 (70.6%)	5 (29.4%)	17 (100%)
Total		145 (67.8%)	69 (32.2%)	214 (100%)

Being a Muslim majority community, 93% of families were Muslim (Hindu: 2.8%, Christians: 4.2%). A hundred and twenty-two, that is, more than half of the interviewees (56.7%) had no formal education and an almost similar number of families (56.4%) had an average monthly income less than 16,000 Pakistani Rupees.

Children presented with various signs and symptoms of CNS infections, the most common being fever in 185 subjects (86%), a sick child in 124 cases (57.9%) and repeated fits in 107 subjects (50%) (Table 2). The vast majority, that is, 177 children (82.3%) concomitantly had other symptoms such as chest infections, vomiting, loose stools, difficulty in walking/speaking, poor feeding, rash, measles, cyanosis or a history of trauma.

**Table 2:** Frequency of presenting signs and symptoms

Subjects’ Presenting Signs and Symptoms	Frequency
Fever	86.0%
Sick Child	57.9%
Repeated fits	50.0%
One episode of fits	48.8%
Age less than 12 months	29.4%
Hypertonia/hyperreflexia	27.4%
Altered level of consciousness	26.0%
Drowsiness	19.1%
Irritability	18.6%
Headache	12.1%
Bulging fontanelle	9.8%
Upgoing plantars	7.0%

Parents and relatives of these subjects were asked if they had been properly counseled by their physicians before taking the decision to have an LP done or not. The indications for performing an LP were explained to 162 families (75.3%). However, the potential complications of the procedure were communicated to only 100 families (46.5%).

It was observed that when indications for performing an LP were appropriately explained, there was a significantly greater number of consents given as compared to when they were not made clear (p=0.009) (Table 3). On the other hand, detailing the complications of the procedure did not considerably impact the decision to refuse LP (p=0.1).

**Table 3:** Counselling of attendants and lumbar puncture (LP) status

		Indications for LP explained?		Total
		Yes	No	
Lumbar Puncture Status	Consent Given	117 (80.7%)	28 (19.3%)	145 (100%)
	Refusal for LP	45 (64.3%)	25 (35.7%)	70 (100%)
Total		162 (75.3%)	53 (24.7%)	215 (100%)

Attendants were also questioned regarding their knowledge and opinion about the potential complications that could follow an LP. Paralysis of lower limbs (n=73, 48.7%) and back pain (n=45, 30%) were mostly seen as complications of LP, while 66 families, i.e., almost a third (30.7%) did not possess any knowledge regarding the risks of the procedure. Few (n= 21, 9.8%) believed that an LP could also cause weakness, blindness, deafness, aphasia, urinary/fecal incontinence, fever, loss of ability to sit and further worsening of the condition.

Parents who had given consent for LP were asked the reason behind their consent and, similarly, where LP was refused, the reasons behind this decision were also inquired. The most common reasons for giving consent were to allow the physicians to make the correct diagnosis in 55.9% of cases (n=80) and to comply to their physician’s advice in 36.9% of cases (n=53); whereas, the reasons for refusal to consent included fear of paralysis in 64.2% of cases (n=43) and fear of death in 31.3% (n=21) (Table 4).

**Table 4:** Parents perception about lumbar puncture (LP) consent and reasons for refusal and consent

Parents’ beliefs about complications of LP	
Paralysis of Lower limbs	48.7%
Don’t know about any complication	30.2%
Back pain	30%
Mental Retardation	14.7%
Trauma to spinal cord	11.3%
Bleeding	10%
Death	9.3%
Infection	7.3%
Infertility	3.3%
Reasons for LP Consent	
To allow correct diagnosis	55.9%
Following physician’s advice	36.9%
Early improvement	22.2%
Better treatment	16%
Fear of physician’s bias	10.5%
Prevention of complications	4.9%
Fear of treatment/admission termination	4.2%
LP considered therapeutic	2.1%
­­­Reasons for LP Refusal	
Fear of paralysis	64.2%
Fear of death	31.3%
Consider LP unnecessary	19.4%
Know someone who died after LP	14.9%
Fear of trauma	12.1%
Fear of infections	4.5%

The multi-variable logistic regression analysis showed that indications of LP not explained to the family, primary/secondary education level (versus uneducated and matric or above), and female caregiver approached for consent increases the likelihood of refusal to LP in our sample (Table 5). The logistic regression model was statistically significant, χ2 (8) = 38.2 p < 0. 001. The model explained 23% (Nagelkerke R2) of the variance in refusal to LP and correctly classified 77% of cases. Gender and the age of the baby, history of fits, and explaining complications of LP to the caregivers were not found predicting the LP refusal in our sample. 

**Table 5:** Multivariable logistic regression analysis showing risk factors for refusal to pediatric lumbar puncture (n=215) LP: Lumbar puncture, B: Slope, χ2: Chi-square value of the model, P: p-value of odds ratio, OR: Odds ratio.

Characteristics	Β	χ2	P	OR	95% CI
Indication of LP explained	1.02	5.1	0.025	2.8	1.1, 6.8
Education level of the Consenter	1.54	17.2	0.000	4.7	2.3, 9.6
Gender of the Consenter	0.72	4.2	0.04	2.0	1.0, 4.1

## Discussion

The term ’informed consent’ focuses on improving the standard of the subjects’ knowledge as well as giving them their due right to authorize or decline participation or intervention [5]. Unfortunately, this involves numerous obscurities. Although generally, parents and guardians wish for good health and wellbeing of their children, their decision is influenced by their opinion about the proposed management of the child’s condition, as well as by their personal beliefs. These, in turn, are shaped by religious, social and cultural values, as well as poverty and illiteracy, especially in a family-centered, community based, multi-cultural society like that of Pakistan [6]. The procedure of LP in children is an example of this delicate matter.

LP allows the examination of cerebrospinal fluid (CSF), which is sometimes essential for the diagnosis of CNS infection, and it is commonly performed in the pediatric population. Despite its effectiveness as a diagnostic procedure, parental refusal to consent to LP has been a subject of ongoing discussion in various parts of the world, which reflects the universal nature of this problem [7-9]. 

High levels of illiteracy, the stigmata associated with the procedure, and an exceedingly family-centered society are all thought to be hindrances for LP consent in Pakistan. This study confirms the high incidence of LP refusal, with almost a third of families (32.6%) declining this intervention. Comparing this with literature from Malaysia, where the refusal rate was 24.7% [8], Saudi Arabia (44.3%) [9], and the UAE (44%) [2], it is apparent in this variation that LP refusal is most likely multi-factorial, involving both physician and patient factors.

Given Pakistan’s patriarchal society where the money-earning male usually makes these important decisions, it was unsurprising to find that the fathers of subjects were the sole decision makers in 61.9% of cases. This finding was however inconsistent with literature from other similar societies where, in the majority of cases, both parents were involved in decision-making, such as in KSA (66%) and the UAE (67%) [8-9]. Another varying detail found in our study was that fathers were more likely to consent to LP than mothers, contrary to other literature where the opposite was observed [7].

One of the aims of the study was to associate education with the frequency of LP consent. This study shows that parents with no formal education consented more readily than those who had received some (primary and secondary) formal education. Although more than half of the parents were uneducated, the refusal rate (32.6%) was still lower compared to a hospital in the UAE, where a study shows a refusal rate of 44% despite the majority of guardians’ having had received secondary school education [7]. The authors' reason that this could be because of the general notion in this culture that physicians are instruments of God (Maseeha) who seek the betterment of their patients, as well as fear of lack of optimal treatment if LP is refused. This study did not reveal any significant difference in the age of guardians, ethnicity, employment status or gender of the patient between families that gave consent and those that did not. The finding is consistent with related literature [7-9]. Similarly, this study also showed the most common signs and symptoms of LP candidates to be fever, repeated fits and sick looking children.

The most common reason for refusing to give consent for LP was found to be fear of paralysis, as seen in as many as 43 cases (64.2%), while 21 families (31.3%) stated their reason for the refusal to be fear of death following the procedure. Few (n=10, 14.9%) had previously seen or heard of cases where someone had died after an LP, while 13 families (19.4%) did not consider LP necessary for the diagnosis/treatment of their child. Paralysis of lower limbs (48.7%), back pain (30%), mental retardation (14.7%), trauma to the spinal cord (11.3%) and death (9.3%) were commonly considered as complications of LP by the general public. These findings were similar to those found in literature, which shows that a comparable number of respondents refused LP as they considered it unnecessary (21%), while 75% of families refused consent due to fear of complications; the vast majority of whom (58%) feared paralysis and 16% feared pain [7]. Similarly, a study in Kuwait also indicates that parents who considered LP unsafe mostly feared paralysis (49.2%) and pain (16.6%) following the procedure [1].

Our study indicates that those who gave consent mostly wanted to know the exact diagnosis (55.9%) and sought early improvement of their child (22.2%), while many simply wished to follow their physician’s advice (36.9%). A similar study assessing the knowledge of all the parents asked for LP consent regarding the procedure reveals that the majority and a quite comparable percent (57.1%) consider diagnosis as the main reason for performing it [1]. Another study conducted in the UAE revealed an interesting datum unalike our findings, that a major determining factor in those that gave LP consent was more awareness of the complications that may follow bacterial meningitis which, in turn, made it easier for families to allow LP and avoid these complications [2].

It was alarming to see that when taking parental permission for LP by the concerned physician, more than half (53.5%) of parents were not told the complications that might follow the procedure. This is not only an ethical indiscretion, but reflects the inefficiency of practitioners in this particular area. This study strongly supports a survey conducted amongst general practitioners in Pakistan, which revealed that though they did feel that their patient has the right to know, a large fraction of them did not deem it necessary to share the details of the proposed intervention to their patients [10]. This would be the right time to point out the vital need to incorporate medical ethics into undergraduate as well as postgraduate curricula. While some may argue that the incidence of a major complication following an LP is too insignificant to mention and discussing these complications may increase the frequency of LP refusal in patients with suspected CNS infection, this study negates this reasoning. It was observed that detailing the potential complication of LP did not have any significant effect of the rate of refusal.

On the other hand, explaining the indications of the procedure (75.3%) did appear to positively impact the consent rate, with families that were detailed the purpose of LP consenting more readily. 

Refusing LP not only harms the patient, but also puts the doctor in a difficult position. As of yet, there is no alternative to LP for making a firm diagnosis of a CNS infection; cultures of CSF samples being considered ‘gold’ standard whereas the newer molecular methods performed on CSF samples considered ‘platinum’ standard [11]. Blood cultures, although valuable if CSF cultures are unavailable or negative, have been shown to yield negative results in 15%-38% of infants with confirmed meningitis [12-15]. In fact, a survey showed that only 62% of blood cultures were concomitantly positive in the presence of CSF culture-positive neonatal meningitis [2], the yield further decreasing by 20% for pre-treated patients, as seen in two studies [16-17]. In this setup, the only other option left is to treat the suspected infection empirically. In doubtful cases, the risks of an untreated CNS infection must be weighed against the risks of admission and administration of intravenous antibiotics and causing burden to a healthcare system [18-20].

## Conclusions

In conclusion, the frequency of LP refusal is high in our setup, and puts practitioners in a difficult position when a suspected CNS infection cannot be confirmed. However, patiently educating parents about the safety and efficacy of the procedure, explaining the indication and need for the procedure, and understanding their perceptions and fears may bring about a change in their decision.
